# The Impact of the COVID-19 Pandemic on the Uptake of Influenza Vaccine: UK-Wide Observational Study

**DOI:** 10.2196/26734

**Published:** 2021-04-14

**Authors:** Patrik Bachtiger, Alexander Adamson, Ji-Jian Chow, Rupa Sisodia, Jennifer K Quint, Nicholas S Peters

**Affiliations:** 1 National Heart & Lung Institute Imperial College London London United Kingdom

**Keywords:** COVID-19, influenza, vaccination, COVID, Pandemic, National Health Service, Health Service, flu, virus, vaccine, impact, uptake, observational, United Kingdom, public health, intention, electronic health record

## Abstract

**Background:**

In the face of the COVID-19 pandemic, the UK National Health Service (NHS) extended eligibility for influenza vaccination this season to approximately 32.4 million people (48.8% of the population). Knowing the intended uptake of the vaccine will inform supply and public health messaging to maximize vaccination.

**Objective:**

The objective of this study was to measure the impact of the COVID-19 pandemic on the acceptance of influenza vaccination in the 2020-2021 season, specifically focusing on people who were previously eligible but routinely declined vaccination and newly eligible people.

**Methods:**

Intention to receive the influenza vaccine in 2020-2021 was asked of all registrants of the largest electronic personal health record in the NHS by a web-based questionnaire on July 31, 2020. Of those who were either newly or previously eligible but had not previously received an influenza vaccination, multivariable logistic regression and network diagrams were used to examine their reasons to undergo or decline vaccination.

**Results:**

Among 6641 respondents, 945 (14.2%) were previously eligible but were not vaccinated; of these, 536 (56.7%) intended to receive an influenza vaccination in 2020-2021, as did 466 (68.6%) of the newly eligible respondents. Intention to receive the influenza vaccine was associated with increased age, index of multiple deprivation quintile, and considering oneself to be at high risk from COVID-19. Among those who were eligible but not intending to be vaccinated in 2020-2021, 164/543 (30.2%) gave reasons based on misinformation. Of the previously unvaccinated health care workers, 47/96 (49%) stated they would decline vaccination in 2020-2021.

**Conclusions:**

In this sample, COVID-19 has increased acceptance of influenza vaccination in previously eligible but unvaccinated people and has motivated substantial uptake in newly eligible people. This study is essential for informing resource planning and the need for effective messaging campaigns to address negative misconceptions, which is also necessary for COVID-19 vaccination programs.

## Introduction

To date, the COVID-19 pandemic has led to over 100,000 deaths in the United Kingdom alone. With increasing regional outbreaks [1], substantial concern has been raised about preparedness for a nationwide escalation of cases throughout winter pressures in 2020-2021 [2-4]. Seasonal influenza places the UK National Health Service (NHS) under considerable pressure each winter, with up to 18,000 additional daily emergency admissions [5] and >4000 hospital beds occupied daily by patients with influenza in 2017-2018 [6,7].

For this reason, the NHS has extended its free seasonal influenza vaccination program for the current season to all people aged over 50 years (previously 65 years) and to include the 11-12 years age group (previously 2-10 years) [8]; thus, an estimated 32.4 million people (48.8% of the UK population) are now eligible [9]. In England in 2019, uptake of the influenza vaccine among those eligible was only 70.6% [10], below the critical 75% target for effectiveness recommended by the World Health Organization [11]. Against a background of declining numbers over the last decade (from a peak of 74.2% in 2008-2009), the uptake this season is not only unknown but is also completely unpredictable. The threat of COVID-19 and the associated publicity educating the public about viruses and vaccine development, coupled with recent evidence that coinfection with influenza and SARS-CoV-2 doubles mortality compared with infection with SARS-CoV-2 alone [12] and that the influenza vaccination may reduce incidence of life-threatening COVID-19 disease in people aged over 65 years [13], are likely to affect attitudes and the public health imperative of mass uptake. With substantial concerns that higher earlier uptake of influenza vaccination in 2020-2021 will rapidly deplete stocks (as already reported [14]), there is still a risk that a lack of informed planning will result in failure to meet the requirements of this public health initiative.

Therefore, the objective of this study was to measure the impact of the COVID-19 pandemic on the acceptance of influenza vaccination in the 2020-2021 season, specifically focusing on people who were previously eligible (aged over 65 years or having an eligible comorbidity) who routinely decline vaccination and newly eligible people (aged 50-64 years)—two groups in which the determinants of vaccine hesitancy may differ. These groups include those at highest risk from COVID-19; if the influenza vaccine confers a reduced risk of COVID-19, understanding specific covariates that relate to vaccine hesitancy can inform public health messaging to maximize uptake and help contend with potential double winter pandemics of influenza and COVID-19.

## Methods

### Ethical Approval

The weekly questionnaire was a direct care tool for patients to self-monitor their well-being during the COVID-19 pandemic. Participants were not paid or otherwise compensated for completing questionnaires. Upon review, the Imperial College Healthcare NHS Trust Data Protection Office advised that ethical approval for data analysis and publication was not required. Participants gave informed consent within the CIE, were free to opt out of receiving questionnaires at any time, and were informed prior to completing their responses that these would be fully anonymized and stored on secure servers before analysis toward informing local and national health policy.

### Study Participants

Participants were registrants of the Care Information Exchange (CIE) of Imperial College Healthcare NHS Foundation Trust. The CIE is the largest patient-facing electronic health record in the United Kingdom; it is accessible by email registration for any patient who has had an encounter at the Trust (UK-wide population, Figure S1 in Multimedia Appendix 1). On June 5, 2020, the CIE held 57,056 registrants, of whom 34,502 were active users, defined as having one or more logins in the preceding month.

Participants in this study were CIE registrants receiving weekly web-based questionnaires through the platform, starting April 9, 2020 (week 1), as a direct care tool for self-monitoring physical, mental, and social well-being during the COVID-19 pandemic. This was the first ever such use of the CIE platform, prompted by the immediate public health priorities to provide patients with a tool to track their well-being and inform local and national health policy through this exercise in participatory epidemiology.

### Questionnaire Design and Timing

A questionnaire including items on the government's expanded influenza vaccination program was sent to participants on July 31, 2020 (week 16, Table S1 in Multimedia Appendix 1). Applying recommendations for questionnaire design [15,16], the question items were developed by a collaboration of experts in qualitative research at Imperial College London, encompassing public health, respiratory epidemiology, and digital health, and were also informed by previous studies [17,18]. Question items were externally peer-reviewed and tested on lay persons (n=5) before being included. The focus was on previous uptake of influenza vaccination, being for or against vaccination in 2020-2021 and reasons why (unrestricted free text responses), health worker status, and presence of school-age children in the household. Responses from participants regarding the presence of school-age children in their household were also recorded. Specifically, they were asked whether they would want any of these children to receive the influenza vaccination if it were offered in 2020-2021. It could not be assumed that people who were vaccinated in the previous year would continue this habit. Subsequently, a specific question was posed to also measure if any participants who were vaccinated in 2019-2020 would not be vaccinated again in 2020-2021.

Responses to items in prior questionnaires in the series were used to complete information on participant ethnicity, additional vaccine eligibility criteria (including chronic disease), index of multiple deprivation (IMD) quintile (obtained from participant postcode), health care utilization since the beginning of the lockdown, whether the participant considered themselves at high risk from COVID-19, experience of any COVID-19 symptoms, self-reported understanding of government advice, anxiety related to a return to lockdown, and whether the participant would agree to receive a COVID-19 vaccine if available.

### Inclusion and Exclusion Criteria

**Figure 1:**
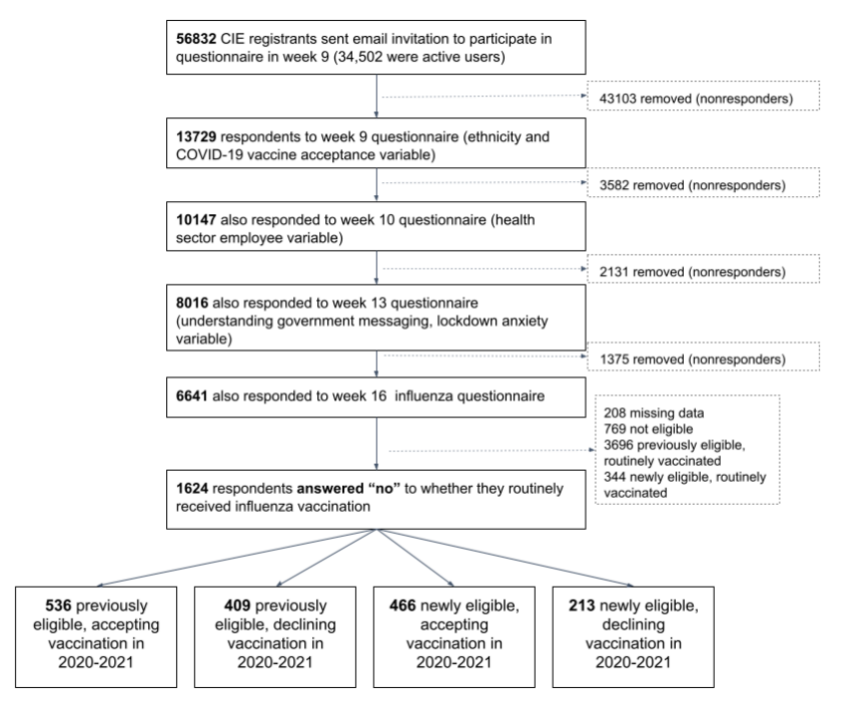
Participant inclusion flow diagram based on responses to questionnaires capturing variables required for analysis. CIE: Care Information Exchange.

Participants were aged 18 years or above and were required to have answered questionnaires capturing variables relevant to the analysis (see the flow diagram in Figure 1) and to have answered “no” to a question assessing whether they routinely received influenza vaccination. Respondents not eligible for influenza vaccination (ie, aged <50 years) were excluded. Participants who submitted incomplete or inconsistent responses to the questions on influenza vaccination were excluded, as were those who answered “prefer not to say” for ethnicity and who were missing responses for other variables required in the analysis, with the exception of postcode. Responses submitted later than 4 days from the time of the questionnaire launch were excluded.

### Definition of Study Groups

The analyses in this study were confined to participants who were eligible for a free NHS influenza vaccination in 2020-2021 who indicated they had previously not routinely received it (this group is the greatest unknown factor when planning resourcing and targeting public health campaigns to maximize uptake). Members of this previously unvaccinated group were either previously eligible (main criteria up to 2019-2020 were age over 65 years, eligible comorbidity, and working in the health care sector) or newly eligible for the expanded 2020-2021 program (age over 50 years).

Further stratification according to whether or not the influenza vaccine would be accepted in 2020-2021 generated four groups: (1) Previously eligible, newly responding “yes,” (2) previously eligible, still responding “no,” (3) newly eligible, responding “yes,” and (4) newly eligible, responding “no.” Owing to inherent differences in age and comorbidity status of the previously and newly eligible cohorts, their covariates for willingness to receive the influenza vaccine may be different; therefore, this stratification was maintained throughout our analyses.

### Data Analysis

Age was categorized into bands of 18-29, 30-39, 40-49, 50-59, 60-69, and 70+ years to enable easier interpretation of a potential nonlinear relationship between age and responses to influenza vaccination. The 10-point scale measurements of “anxiety related to return to lockdown” and “understanding of government messaging” were regrouped into categories of 1-2, 3-4, 5-6, 7-8, and 9-10, and ethnicity was categorized into five groups due to low numbers in some categories. Descriptive statistics reported for the data set are broken down according to study group. Differences in categorical variables were assessed by chi-square test or by Fisher exact test where chi-square test assumptions were violated, and differences in continuous variables were assessed using *t* tests. *P* values <.05 were considered statistically significant.

The effects of variables of interest on the inclination to receive an influenza vaccination were calculated using univariate and multivariable logistic regression models, with presentation of both to identify if results in the univariate analysis were due to confounding by other collected variables. The relationship between age (the only continuous variable) and the log odds of receipt of influenza vaccination were plotted and visually inspected. If the effect appeared to be linear, age was included as a linear variable; otherwise, it was included as a categorical variable. All data were analyzed in R, version 3.6.2 (R Project). Variables with low numbers in categories were not included in the multivariable analyses. “Acceptance of COVID-19 vaccine if available” was deemed likely to be highly correlated with “accepting influenza vaccine in 2020-2021” and was not included in multivariable models to enable greater interpretation of other predictors. Multicollinearity was assessed by calculation of the variance inflation factor (VIF), and variables with a VIF >5 (indicating substantial multicollinearity) were removed from the model.

Each participant not routinely receiving influenza vaccination, whether previously or newly eligible, was asked to qualify their yes/no response to whether they would accept vaccination in 2020-2021 using a free text response option. Three researchers, blinded to the responses on vaccine acceptance, each independently coded the content of 100 responses according to multiple prospectively identified themes that could co-occur. A consensus was then reached to define the main themes for coding the remaining responses. For example, “I don’t see the point because I’ve never had flu [influenza]” was coded as “unnecessary” and “not had flu before.” A full list of the themes with examples is available in Table S2 in Multimedia Appendix 1.

Using this codified qualitative data, a network diagram [19,20] was generated for each of the four groups using the Networkx package in Python, version 3.7. Dimensions of centrality and overall topography of the nodes were not applicable; thus, the network was laid out in a comprehensible circular “shell” arrangement. Each diagram was limited to the 10 most represented themes within each group’s responses. Nodes were color-coded to reflect positive, negative, and neutral sentiments of the themes. Separately, reasons for health care workers’ continued nonvaccination in 2020-2021 were reported descriptively.

### Dissemination to Participants and Related Patient and Public Communities

We plan to disseminate the findings of this study to participants in the Imperial College Healthcare NHS Trust’s annual web-based newsletter.

## Results

### Sample Characteristics

Among respondents aged ≥18 years, 6641 completed the week 16 questionnaire on influenza vaccination in the predefined time period and the requisite previous questionnaires to complete the baseline characteristics (Figure 1). Of these, 208 (3.1%) were missing answers to one or more essential variables and were removed, leaving 6433 complete responses. The total number of previously eligible but unvaccinated (n=945) and newly eligible but unvaccinated (n=679) participants was 1624 (see Figure 1 for details).

Of the vaccinated and unvaccinated previously eligible participants, those who had previously declined vaccination were more likely to be younger (median age 61 years, IQR 51-67, vs median age 67 years, IQR 58-73, *P*<.001), female (520/945, 55.0%, vs 1727/3696, 46.7%, *P*<.001), have chronic neurological disease (102/945, 10.8%, vs 241/3696, 6.5%, *P*<.001), work in the health sector (96/945, 10.2%, vs 287/3696, 7.8%, *P*=.02), and be in a lower IMD quintile (*P*=.03), and they were less likely to have chronic respiratory disease (137/945, 14.5%, vs 757/3696, 20.5%, *P*<.001) or chronic heart disease (66/945, 7.0%, vs 757/3696, 12.2%, *P*<.001) compared to those who were previously eligible and received the vaccine. Of the newly eligible participants, when compared with those who had received the vaccine despite being ineligible by NHS criteria, those who had not received the vaccine were more likely to be younger (mean age 57 years, IQR 54-61, vs median age 59 years, IQR 55-63) and in a lower IMD quintile (Table S3, Multimedia Appendix 1). Among all respondents who indicated having received the influenza vaccine in 2019-2020, 309/6867 (4.5%) responded that they did not intend to repeat this in 2020-2021.

### Change in Acceptance and Uptake of Influenza Vaccine in 2020-2021

Summary statistics for the groups broken down according to vaccine eligibility and acceptance of the influenza vaccine in 2020-2021 are shown in Table 1. Of those previously eligible but routinely not vaccinated, 536 (56.7%) intended to be vaccinated in 2020-2021, increasing the vaccination rate in the entire previously eligible cohort from 79.6% to 91.2%. Among the newly eligible, 466 (68.6%) reported they would accept vaccination in 2020-2021.

**Table 1:** Characteristics of the study participants (N=1624) based on UK-wide responses to web-based questionnaires administered through Care Information Exchange (influenza-related questionnaire sent July 31, 2020). Baseline demographics and questionnaire responses of all participants previously not routinely receiving influenza vaccination are grouped by previously eligible but nonvaccinated and newly eligible, further stratified by acceptance (yes/no) of influenza vaccination in 2020-2021.

Characteristic		Value			
		Previously eligible and plans to receive the influenza vaccine (n=536, 56.7%)	Previously eligible and does not plan to receive the influenza vaccine (n=409, 43.3%)	Newly eligible and plans to receive the influenza vaccine (n=466, 68.6%)	Newly eligible and does not plan to receive the influenza vaccine (n=213, 31.4%)
Age, median (IQR)		62.0 (51.0-67.0)	60.0 (49.0-68.0)	58.0 (55.0-61.8)	56.0 (53.0-60.0)
**Sex, n (%)**					
	Male	248 (46.3)	177 (43.3)	214 (45.9)	69 (32.4)
	Female	288 (53.7)	232 (56.7)	252 (54.1)	144 (67.6)
**Ethnicity, n (%)**					
	White	453 (84.5)	320 (78.2)	415 (89.1)	181 (85.0)
	Asian	36 (6.7)	39 (9.5)	19 (4.1)	13 (6.1)
	Black	15 (2.8)	20 (4.9)	13 (2.8)	9 (4.2)
	Mixed	8 (1.5)	6 (1.5)	6 (1.3)	2 (.9)
	Other	24 (4.5)	24 (5.9)	13 (2.8)	8 (3.8)
Eligible disease, n (%)		368 (68.7)	282 (68.9)	N/A^a^	N/A
Chronic respiratory disease, n (%)		71 (13.2)	66 (16.1)	N/A	N/A
Chronic heart disease, n (%)		40 (7.5)	26 (6.4)	N/A	N/A
Chronic kidney disease, n (%)		25 (4.7)	23 (5.6)	N/A	N/A
Chronic liver disease, n (%)		15 (2.8)	11 (2.7)	N/A	N/A
Chronic neurological disease, n (%)		48 (9.0)	54 (13.2)	N/A	N/A
Immunocompromised, n (%)		196 (36.6)	137 (33.5)	N/A	N/A
Other eligible comorbidity, n (%)		103 (19.2)	93 (22.7)	N/A	N/A
Health sector employee, n (%)		47 (8.8)	49 (12.0)	N/A	N/A
**Index of multiple deprivation, n (%)**					
	1	34 (6.3)	31 (7.6)	17 (3.6)	14 (6.6)
	2	78 (14.6)	64 (15.6)	69 (14.8)	36 (16.9)
	3	107 (20.0)	59 (14.4)	94 (20.2)	29 (13.6)
	4	85 (15.9)	55 (13.4)	87 (18.7)	28 (13.1)
	5	79 (14.7)	44 (10.8)	57 (12.2)	15 (7.0)
	Missing	153 (28.5)	156 (38.1)	142 (30.5)	91 (42.7)
**Health care utilization, n (%)**					
	None	91 (17.0)	89 (21.8)	145 (31.1)	80 (37.6)
	Any	445 (83.0)	320 (78.2)	321 (68.9)	133 (62.4)
Considers self at high risk from COVID-19, n (%)		346 (64.6)	267 (65.3)	140 (30.0)	41 (19.2)
**Understanding of government messaging (score from 1-10)** **, n (%)**					
	1-2	31 (5.8)	34 (8.3)	48 (10.3)	16 (7.5)
	3-4	69 (12.9)	48 (11.7)	65 (13.9)	24 (11.3)
	5-6	138 (25.7)	93 (22.7)	107 (23.0)	53 (24.9)
	7-8	190 (35.4)	133 (32.5)	159 (34.1)	72 (33.8)
	9-10	108 (20.1)	101 (24.7)	87 (18.7)	48 (22.5)
**Anxiety related to return to lockdown (score from 1-10)** **, n (%)**					
	1-2	87 (16.2)	85 (20.8)	76 (16.3)	50 (23.5)
	3-4	90 (16.8)	59 (14.4)	85 (18.2)	35 (16.4)
	5-6	149 (27.8)	111 (27.1)	127 (27.3)	48 (22.5)
	7-8	150 (28.0)	105 (25.7)	130 (27.9)	50 (23.5)
	9-10	60 (11.2)	49 (12.0)	48 (10.3)	30 (14.1)
**Acceptance of COVID-19 vaccine if available** **, n (%)**					
	Not sure	100 (18.7)	159 (38.9)	72 (15.5)	85 (39.9)
	No	35 (6.5)	117 (28.6)	25 (5.4)	38 (17.8)
	Yes	401 (74.8)	133 (32.5)	369 (79.2)	90 (42.3)

^a^N/A: not applicable.

### Predictors of Willingness to Receive Influenza Vaccination

In the univariate analysis (Tables 2 and 3), willingness to receive a COVID-19 vaccine was associated with willingness to receive an influenza vaccination in 2020-2021 in both groups compared to those who were unsure (odds ratio [OR] 4.79, 95% CI 3.50-6.61, vs OR 4.84, 95% CI 3.29-7.17). Among respondents who would newly accept influenza vaccination, of those who were previously eligible and newly eligible, 401/536 (74.8%) and 369/466 (79.2%), respectively, responded they would accept a COVID-19 vaccination, compared to 133/409 (32.5%) and 90/213 (42.3%) of those declining the influenza vaccine.

In respondents who were previously eligible, answering “no” in response to whether they would receive a COVID-19 vaccination if offered was associated with a lower likelihood of wanting to receive the influenza vaccination in 2020-2021 (OR 0.48, 95% CI 0.30-0.74), as was having a chronic neurological disease (OR 0.65, 95% CI 0.43-0.98). Although people aged 60-69 years were more likely to respond “yes” than those aged ≥70 years (OR 1.48, 95% CI 1.02-2.14), no clear effect of age was found in people below the age of 60 years. The multivariable analysis (Tables 2 and 3) resulted in few substantial changes to effect estimates, with the exception of age, for which all estimates shifted upward (showing a stronger association with an increased likelihood of answering “yes” after adjustment for other variables).

**Table 2:** Unadjusted and adjusted logistic regressions to predict a “yes” response for participants who would accept an influenza vaccine in 2020-2021 and who were previously eligible but did not routinely receive influenza vaccination.

Characteristic		Unadjusted odds ratio (95% CI)	Adjusted^a^ odds ratio (95% CI)
**Age (years; reference category: ≥70)**			
	18-29	1.99 (0.85-5.06)	2.53 (1.00-6.89)
	30-39	0.83 (0.46-1.49)	1.20 (0.62-2.31)
	40-49	0.86 (0.54-1.35)	1.17 (0.70-1.95)
	50-59	1.11 (0.75-1.66)	1.42 (0.90-2.25)
	60-69	1.48 (1.02-2.14)	1.61 (1.09-2.37)
Female sex		0.89 (0.68-1.15)	0.93 (0.70-1.23)
**Ethnicity (reference category: White)**			
	Asian	0.65 (0.40-1.05)	0.71 (0.43-1.18)
	Black	0.53 (0.26-1.05)	0.58 (0.27-1.18)
	Mixed	0.94 (0.32-2.89)	1.09 (0.36-3.50)
	Other	0.71 (0.39-1.27)	0.71 (0.39-1.31)
**Comorbidity**			
	Chronic respiratory disease	0.79 (0.55-1.14)	0.78 (0.52-1.18)
	Chronic heart disease	1.19 (0.72-2.00)	1.03 (0.60-1.79)
	Chronic kidney disease	0.82 (0.46-1.48)	0.71 (0.38-1.33)
	Chronic liver disease	1.04 (0.48-2.35)	0.94 (0.41-2.22)
	Chronic neurological disease	0.65 (0.43-0.98)	0.62 (0.38-0.99)
	Immunocompromised	1.14 (0.87-1.50)	0.95 (0.69-1.32)
	Other comorbidity	0.81 (0.59-1.11)	0.79 (0.56-1.10)
Health sector employee		0.71 (0.46-1.08)	0.76 (0.46-1.24)
**Index of multiple deprivation quintile (reference category: 1)**			
	2	1.11 (0.62-2.00)	1.09 (0.59-2.01)
	3	1.65 (0.92-2.96)	1.54 (0.84-2.82)
	4	1.41 (0.78-2.55)	1.29 (0.70 to 2.40)
	5	1.64 (0.89 to 3.02)	1.59 (0.84-3.00)
	Missing	0.89 (0.52-1.53)	0.91 (0.52-1.59)
Health care utilization		1.36 (0.98-1.88)	1.41 (0.99-2.01)
Considering self at high risk from COVID-19		0.97 (0.74-1.27)	1.03 (0.76-1.39)
**Understanding of government messaging (score from 1-10; reference category: 5-6)**			
	1-2	0.61 (0.35-1.07)	0.59 (0.33-1.05)
	3-4	0.97 (0.62-1.53)	0.89 (0.56-1.43)
	7-8	0.96 (0.68-1.36)	0.92 (0.64-1.31)
	9-10	0.72 (0.49-1.05)	0.75 (0.50-1.11)
**Anxiety related to return to lockdown (score from 1-10; reference category: 5-6)**			
	1-2	0.76 (0.52-1.12)	0.90 (0.60-1.37)
	3-4	1.14 (0.76-1.72)	1.14 (0.74-1.75)
	7-8	1.06 (0.75-1.51)	1.07 (0.74-1.54)
	9-10	0.91 (0.58-1.43)	1.06 (0.66-1.71)
**Acceptance of COVID-19 vaccine if available (reference category: “unsure”)**			
	No	0.48 (0.30-0.74)	N/A^b^
	Yes	4.79 (3.50-6.61)	N/A

^a^Adjusted odds ratios were adjusted for every other variable in the model (age, sex, ethnicity, disease, index of multiple deprivation quintile, health care utilization, considering oneself at high risk for COVID-19, undertaking any COVID-19 test, believing oneself to have had COVID-19, understanding of government advice, anxiety related to a return to lockdown).

^b^N/A: not applicable.

**Table 3:** Unadjusted and adjusted logistic regressions to predict a “yes” response for participants who would accept an influenza vaccine in 2020-2021 and who were newly eligible and not routinely vaccinated.

Characteristic		Unadjusted odds ratio (95% CI)	Adjusted^a^ odds ratio (95% CI)
Age		1.07 (1.03-1.12)	1.06 (1.01-1.10)
Female		0.56 (0.40-0.79)	0.54 (0.37-0.77)
Asian		0.64 (0.31-1.35)	0.57 (0.26-1.29)
Black		0.63 (0.27-1.55)	0.76 (0.30-2.01)
Mixed		1.31 (0.30-8.99)	0.89 (0.17-6.63)
Other		0.71 (0.29-1.82)	0.77 (0.29-2.17)
Other comorbidity		1.17 (0.79-1.76)	1.01 (0.66-1.59)
**Index of multiple deprivation** **quintile**			
	2	1.58 (0.69-3.57)	1.60 (0.66-3.85)
	3	2.67 (1.17-6.08)	2.51 (1.02-6.13)
	4	2.56 (1.11-5.86)	2.63 (1.07-6.45)
	5	3.13 (1.26-7.86)	2.83 (1.07-7.59)
	Missing	1.29 (0.60-2.73)	1.16 (0.51-2.63)
Health care utilization		1.33 (0.95-1.87)	1.45 (1.00-2.11)
Considering self at high risk for COVID-19		1.80 (1.22-2.70)	2.00 (1.29-3.16)
**Understanding government messaging (score from 1-10)**			
	1-2	1.49 (0.78-2.92)	1.46 (0.73-3.02)
	3-4	1.34 (0.76-2.40)	1.24 (0.68-2.30)
	7-8	1.09 (0.71-1.68)	0.98 (0.62-1.56)
	9-10	0.90 (0.55-1.46)	1.00 (0.59-1.68)
**Anxiety related to return to lockdown (score from 1-10)**			
	1-2	0.57 (0.35-0.93)	0.53 (0.31-0.90)
	3-4	0.92 (0.55-1.54)	0.95 (0.55-1.65)
	7-8	0.98 (0.62-1.57)	0.93 (0.57-1.53)
	9-10	0.60 (0.34-1.07)	0.56 (0.30-1.05)
**Acceptance of COVID-19 vaccine if available**			
	No	0.78 (0.43-1.40)	N/A
	Yes	4.84 (3.29-7.17)	N/A

^a^Adjusted odds ratios were adjusted for every other variable in the model (age, sex, ethnicity, disease, index of multiple deprivation quintile, health care utilization, considering oneself at high risk for COVID-19, undertaking any COVID-19 test, believing oneself to have had COVID-19, understanding of government advice, anxiety related to a return to lockdown).

^b^N/A: not applicable.

In respondents who became newly eligible to receive the influenza vaccine, there was an association between increased age (OR for 1-year increase in age 1.07, 95% CI 1.03-1.12), IMD quintile, and considering oneself at high risk from COVID-19 (OR 1.80, 95% CI 1.22-2.70) and answering “yes” to receiving the influenza vaccine if offered. Female respondents were less likely to answer “yes” (OR 0.56, 95% CI 0.40-0.79), as were those who rated their anxiety about the lifting of lockdown as 1-2 (low anxiety) (OR 0.57, 95% CI 0.35-0.93), compared to those who rated it 5-6. Multivariable analysis resulted in minimal changes to the estimates, demonstrating that the univariate associations found were not due to confounding by the other variables included in the model.

### Subgroup Analyses of Health Care Workers and Households with School-Age Children

In the cohort of previously unvaccinated health care workers (n=96), 49 (51.0%) stated they would accept the influenza vaccine in 2020-2021, compared to 47 (49.9%) who would continue to decline it. The question items pertaining to influenza vaccination of school-age children was answered by 1419/1624 participants (87.4%). Among these, 150/1419 (10.6%) responded that they had school-age children in their household and answered “yes” or “no” to whether they would want any children to be vaccinated in 2020-2021 if offered. Among the 71 participants who were previously eligible but not routinely vaccinated, 33/40 (83%) of those who would accept vaccination in 2020-2021 would also vaccinate their children, compared to 8/31 (26%) of those who would not accept the influenza vaccine for themselves (Fisher exact test, *P*<.001). Among the 79 participants who were previously unvaccinated and newly eligible in 2020-2021, 46/56 (82%) of those who would receive an influenza vaccine this year would want their child to have it also, compared to 10/23 (44%) of those who would not get the influenza vaccine for themselves (Fisher exact test, *P*=.001).

### Network Diagram of Reasons For or Against Vaccination

**Figure 2:**
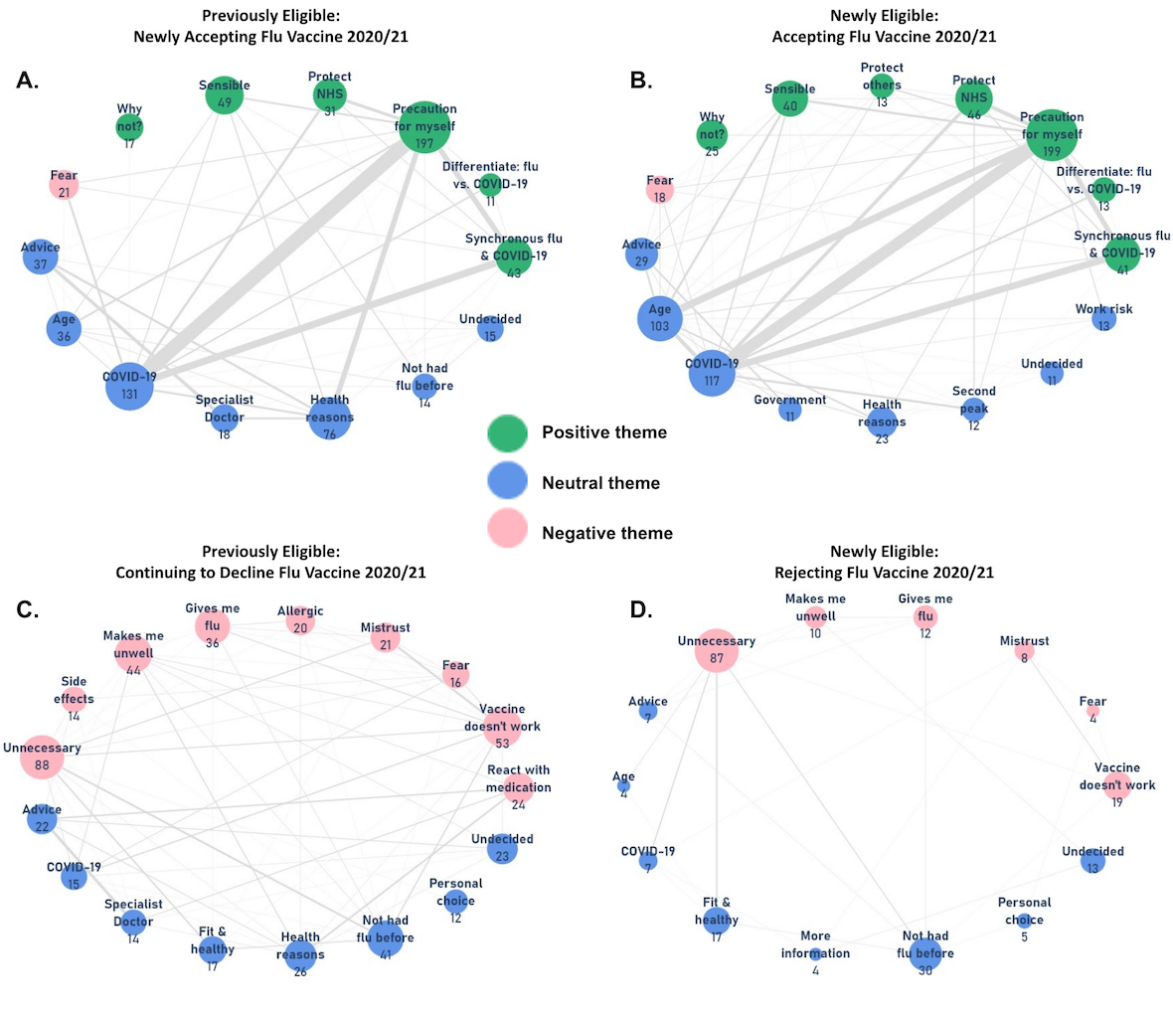
Study participants (N=1624) from UK-wide responses to web-based questionnaires administered through Care Information Exchange (influenza-related questionnaire sent July 31, 2020); network diagram of free-text responses (n=1453, 89.5%). Responses from previously eligible respondents who had previously not accepted the influenza vaccine but would (A) accept it in 2020-2021 (n=478) or (B) continue to decline it (n=356); responses from newly eligible participants who would (C) accept vaccination (n=432) or (D) decline it (n=187). A connecting line (edge) between nodes implies at least one response in which themes of connected nodes co-occurred; the thickness of the line corresponds to the frequency of co-occurrence. Flu: influenza; NHS: National Health Service.

A free text response qualifying why participants would or would not accept influenza vaccination in 2020-2021 was submitted by 834/945 (88.3%) from the previously eligible, unvaccinated group and 619/679 (91.2%) of the newly eligible group. These were coded according to 45 themes (the full list is provided in Table S2 in Multimedia Appendix 1). Figure 2 displays network diagrams for the 10 most common themes for each group.

Among the previously eligible respondents, the three most frequent themes among those newly accepting influenza vaccination in 2020-2021 were “precaution for myself” (197/478, 41.2%), “COVID-19” (131/478, 27.4%), and “health reasons” (76/478, 15.9%); among the newly eligible respondents, the three most frequent themes were “precaution for myself” (199/432, 46.1%), “COVID-19” (117/432, 27.1%) and “age” (103/432, 23.9). “Precaution for myself” was qualified by “COVID-19” in 71/197 (36.0%) and 58/199 (29.1%) participants.

For the previously and newly eligible groups declining vaccination, the three most frequent themes were “unnecessary” (88/356, 24.7%), “vaccine doesn’t work” (53/356, 14.9%), and “makes me unwell” (54/356, 15.2%), and “unnecessary” (87/187, 46.5%), “not had flu before” (30/188, 16.0%) and “vaccine doesn’t work” (19/186, 10.2%), respectively.

### Reasons for Continued Nonvaccination Among Health Care Workers

Of the health care workers reporting previous nonvaccination, 89/104 (85.6%) submitted qualifying responses, among whom 47 were from those newly accepting and 42 continuing to decline vaccination in 2020-2021. For the former, “precaution for myself” (17/47, 36.2%), “COVID-19” (16/47, 34.0%) and “health reasons” (8/47, 17.0%) were the most cited reasons. In those continuing to decline, most frequent reasons were “gives me flu” (10/42, 23.8%), “vaccine doesn’t work” (8/42, 19.0%) and “unnecessary” (6/42, 14.3%).

## Discussion

### Principal Findings

Due to the threat of COVID-19 and the associated publicity educating the public about viruses and vaccine development, following a decade of declining numbers, uptake of the influenza vaccine this year is both unknown and unpredictable. With early reports that higher uptake of influenza vaccination will rapidly deplete stocks [14], there is yet again a threat of a lack of informed planning resulting in failure to meet the demands of a public health initiative. Our findings, including that >90% of previously and 70% of newly eligible participants want vaccination, provide strong evidence to inform planning and public health messaging to maximize vaccination.

The finding that coinfection doubles the risk of death [12] was published after collection of the data described in this study; however, our results indicate that specific avoidance of “synchronous influenza and COVID-19” and “differentiating influenza from COVID-19” were already motivators for new influenza vaccine uptake for the 2020-2021 season. This suggests that the UK public already perceived the risk from a convergence of both viruses. Indeed, in this study, increasing age, IMD quartile, and higher levels of anxiety were associated with increased likelihood of accepting vaccination among the newly eligible; however, the strongest association was considering oneself at high risk from COVID-19, which was associated with an 80% increase in uptake. This relates to our observation in the network diagram that the common reason of “precaution for myself” was frequently qualified by “COVID-19” in both of the groups accepting vaccination. Among those not accepting vaccination, the newly eligible appear to be predominantly motivated by a belief that vaccination is “unnecessary,” contrasting with previously eligible respondents, who gave substantially more misinformed reasons (eg, “gives me flu”), presumably by virtue of having more experience and exposure to vaccination and therefore having more time to develop misinformed beliefs.

Our finding that previously eligible but unvaccinated respondents in the 60-69 years age group were 50% more likely to respond “yes” to vaccination in 2021 than those aged ≥70 years is perhaps unsurprising, given that the latter are at highest risk if exposed—as in, by leaving home to receive an influenza vaccine—to COVID-19. The observation that chronic neurological disease was associated with more vaccine hesitancy may be explained by patients receiving specific therapy (such as for multiple sclerosis) contraindicating influenza vaccination.

Childhood influenza vaccination in the United Kingdom has never reached its 65% uptake target (60.8% in 2018-2019) [21], and our study suggests part of the narrative around unvaccinated children is that adults in their household may also be hesitant to receive an influenza vaccine themselves. Perhaps more concerning is that children may assume their parents’ attitudes to vaccination in later life [19]. Public trust is critical for confidence in vaccination programs [20,22], which must be underpinned by clear messaging campaigns; this is particularly relevant for newly eligible people who, as shown in our study, express fewer misinformed views around the influenza vaccine. Media coverage during the current global health crisis has led to an unprecedented level of education of the general public on respiratory viruses and vaccine development and associated trust in scientific reporting [23,24]. However, social media can potentially be damaging by proliferating misinformation [25]. Collectively, misinformed themes of “makes me unwell,” “gives me flu,” and “vaccine doesn’t work” were present across 35.1% and 20.9% of responses in previously unvaccinated and newly eligible respondents, respectively. Governmental messaging campaigns to address misconceptions such as these are doubly important because they have the potential not only to increase uptake of the influenza vaccine but also to prevent these same misconceptions from undermining the uptake of a future COVID-19 vaccine. Transparency in how a vaccine is being developed must be accompanied by assurances that safety and efficacy are critical and that problematic vaccines will be avoided, which might otherwise diminish public trust [26].

This study suggests that the UK population continues to feel a sense of duty to the NHS; 8.5% of those newly accepting vaccination cited “protect the NHS” as their reason. This messaging, which was used to encourage adherence to the government’s stay-at-home policy during the height of the first wave of the pandemic [27], could also be leveraged to increase uptake of influenza and COVID-19 vaccines. It is noteworthy in the context of the general public’s motivation to protect the NHS that 50% of health care professionals in this sample who previously refused the influenza vaccine still do not intend to receive it. Confirmation of this finding requires further study of larger cohorts of such professionals.

### Limitations

This study has several limitations. These results are only indicative; whether the participants would maintain their responses when faced with influenza vaccination is uncertain. Intentionality may not always translate to actual vaccine uptake. Although one study of US adults aged over 18 years suggested that just over half of respondents who declared intending to receive an influenza vaccine followed through [28], follow-through in the population aged over 50 years in our study is likely to be significantly higher [29]. The advantage of this study using the CIE of the NHS to collect responses is an inherent ability to link to both primary and secondary care data, thereby enabling us to further progress this work at the end of the 2020-2021 influenza season by measuring how intentionality translated to actual uptake.

Use of the CIE, to which all participants were registered, implies both a higher disease burden and better agency over one’s health, and notably, the previously eligible population had a higher baseline uptake (79.6%) than last year’s national average (70.6%). This is more broadly indicative of a sample that is not fully representative of the general population, although our data do suggest that some of the lower IMD quintiles were adequately captured. Despite a representative distribution of questionnaires, ethnic minority groups were underrepresented among the respondents, limiting the generalizability of the acceptance rates and their reasons for and against new uptake. Our study could not fully consider potential mismatches between those eligible for influenza vaccination and those at highest risk of severe COVID-19. By also examining changes in vaccine hesitancy in those ineligible for influenza vaccination but nonetheless at higher risk of COVID-19, such as people who are nonmorbidly obese [30], we could inform policy for further extension of the influenza vaccine criteria to include such individuals. The time-sensitive need to accumulate these data prohibited the generation of question items using, for example, in-depth Delphi methods and full psychometric evaluation of validity; however, an expert team including patient representation designed the questionnaire.

### Conclusion

In the sample in this study, the COVID-19 pandemic has influenced increased acceptance of influenza vaccination in 2020-2021 in people who were previously eligible for the vaccine but routinely unvaccinated, and it is also a major driver of acceptance among people who are newly eligible for the vaccine. This high anticipated demand requires appropriate planning but can be further increased with effective messaging campaigns to address negative misconceptions about influenza vaccination, which may also help prepare for future COVID-19 vaccination. Maximizing vaccination requires informed planning of vaccine supply and public health messaging if we are to avoid failure once again of an essential public health response to the COVID-19 pandemic this winter.

### Data Availability

Imperial College Healthcare NHS Trust is the data controller. The data sets analyzed in this study are not publicly available but can be shared for scientific collaboration subject to meeting requirements of the institution’s data protection policy.

## Appendix

Multimedia Appendix 1 Supplementary materials.

## Abbreviations

CIE: Care Information Exchange
IMD: index of multiple deprivation
NHS: National Health Service
OR: odds ratio
VIF: variance inflation factor
